# Multi-session tDCS over the posterior parietal cortex and associative memory

**DOI:** 10.1371/journal.pone.0318593

**Published:** 2025-01-30

**Authors:** Luka Juras, Marina Martinčević, Uroš Konstantinović, Saša R. Filipović, Andrea Vranić, Jovana Bjekić

**Affiliations:** 1 Department of Psychology, Faculty of Humanities and Social Sciences, University of Zagreb, Zagreb, Croatia; 2 Human Neuroscience Group and Centre for Neuroscience and Neuromodulation, Institute for Medical Research, University of Belgrade, Belgrade, Serbia; University of Utah, UNITED STATES OF AMERICA

## Abstract

Associative memory (AM) plays a crucial role in our ability to link disparate elements of our experiences, yet it is especially vulnerable to age-related decline and pathological conditions. Non-invasive brain stimulation (NIBS), particularly transcranial direct current stimulation (tDCS), has been investigated as a potential intervention to enhance cognitive functions, including AM. Previous tDCS studies yielded inconsistent results, often due to variations in stimulation sites and protocols. Nonetheless, enough evidence suggests that tDCS over the posterior parietal cortex (PPC) can improve AM performance. This study aimed to investigate the cumulative effects of multiple anodal tDCS over the PPC on AM performance alongside item memory and verbal fluency. In a randomized sham-controlled trial, 59 healthy young adults were assigned to either anodal or sham stimulation group, receiving tDCS (1.5 mA, for 20 minutes, at P3) over three consecutive days. Memory performance was assessed at four timepoints: pretest, immediately after the first session, posttest (Day 5), and follow-up (Day 9). Although tDCS was well tolerated, the anticipated enhancement of memory performance was not observed. We interpret these findings in the light of methodological considerations and propose potential explanations for the observed results emphasizing the large between-participants variability in memory performance as a significant factor that may have hindered the detection of tDCS effects.

## Introduction

In our daily lives, we can seamlessly integrate various pieces of information into unique experiences, regardless of their duration, context, modality, or domain. This process of creating cohesive entities from disparate elements is encapsulated in the concept of associative memory (AM). In cognitive neuroscience, AM is defined as the ability to bind previously unrelated pieces of information and store it as a unified representation which is accessible when sought for retrieval [1]. AM is used as an umbrella term for binding-dependent memories, making it an essential building block in the creation of declarative, episodic as well as autobiographical memories. In other words, whether it is recalling a person’s name upon seeing their face or recalling entire episodes from our past triggered by specific scents, we heavily rely on this cognitive ability to navigate our daily lives. However, normative aging and various pathological conditions tend to impair memory function [2, 3]. It appears that AM is particularly vulnerable to age-related decline since older adults exhibit greater deficits in recognizing associated items than single items [4]. Moreover, the AM deficits have been proposed as the potential underlying mechanism of age-related cognitive decline in general [5], which according to the associative deficit hypothesis could be attributed to the decline in automatic binding processes [6]. Hence, enhancing AM represents a significant challenge for researchers and practitioners in cognitive neuroscience and neurorehabilitation. Given the limited efficacy coupled with unfavorable side effects and counterindications profile of pharmacological treatments for memory deficits [7, 8], significant effort has been put into developing and validating non-pharmacological approaches. One line of research has been targeting lifestyle-modification-based interventions, such as physical exercise, dietary modifications, and cognitive training, but with uncertain results yet [9]. Another line of research has been oriented more towards plasticity-based interventions using non-invasive brain stimulation techniques (NIBS). These techniques modulate brain activity through externally applied electrical (transcranial electric stimulation, tES) or magnetic fields (transcranial magnetic stimulation, TMS), and have demonstrated safety, high tolerability, cost-effectiveness, making them a highly relevant and promising path to pursue for practitioners and patients. Among NIBS methods, transcranial direct current stimulation (tDCS) emerges as particularly aligned with these prerequisites [10]. More specifically, tDCS offers several practical advantages over TMS including its lower cost, portability, ease of use, and ability to deliver continuous, low-intensity stimulation over extended durations without risk of inducing seizures [11], allowing for home-based, and remotely supervised clinical applications involving repeated sessions.

The tDCS utilizes a weak electrical current to modulate brain activity, altering neuronal excitability to impact behavioral responses [12]. tDCS operates on the principle of polarity-dependent effects, where anodal stimulation enhances neuronal excitability through depolarization, while cathodal stimulation diminishes it via hyperpolarization of neuronal membranes beneath the electrode and within functionally connected regions of the central nervous system [13]. Moreover, these effects tend to outlast the stimulation period presumably via long-term synaptic plasticity changes. Research on tDCS effects shows that it can induce not only immediate alterations in cortical excitability but also engage changes beyond the stimulation period, mirroring long-term potentiation following anodal stimulation and long-term depression following cathodal stimulation [14].

The hippocampus and surrounding medial temporal lobe are considered the core neural structures involved in AM formation [15], which makes them best candidate site to stimulate in an attempt to enhance AM. However, its anatomical location makes this area unfeasible for direct stimulation since tDCS effects are the strongest on the cortical surface just below the electrodes. To overcome this, stimulating cortical sites with high functional connectivity to the hippocampus was proposed, as cortical activity can propagate to more distant neural areas via functional connectivity networks [16]. For instance, hippocampus has widespread functional networks to frontal, temporal, and parietal cortices [15], all shown to have some contribution to AM [17–19], which makes them viable sites for stimulation. This has led to a considerable variation in electrode montages across studies examining the effects of tDCS on AM performance. A recent systematic review suggests that targeting the posterior parietal cortex (PPC) in healthy young adults generally enhances AM performance, whereas stimulation of frontal regions yields much more inconsistent results [1]. The outcomes disparity can be attributed to the differential activation of neural networks. Specifically, stimulating the PPC engages the parietal-hippocampal functional network, which plays an important role in memory processes [20]. This approach gained support from a series of TMS studies demonstrating the functional importance of the PPC-hippocampus relay in enhancing AM [20–22], Specifically, MRI-guided TMS targeting the posterior parietal cortex (PPC) increased hippocampal activation and strengthened parieto-hippocampal functional connectivity, ultimately leading to improved AM performance [23]. Conversely, stimulating frontal sites and activating the frontal-hippocampal network may primarily modulate other cognitive functions, such as executive functions [24]. Thus, in the case of frontal stimulation, improved AM performance can be possibly viewed as an indirect result of modulating these memory-supportive functions [1].

In a series of experiments [25–27], a single 20-minute session of 1.5 mA anodal tDCS over the PPC yielded immediate and delayed enhancements in participants’ performance on the cued recall AM task. Despite these promising findings, further research is warranted to establish more definitive evidence concerning the impacts of anodal tDCS over the PPC on AM performance. Primarily, these studies utilized a single stimulation session, leaving the cumulative effects of multiple sessions unexplored. Moreover, previous studies assessed the effects on AM only, leaving it unclear whether PPC stimulation selectively influences AM performance or enhances memory performance more broadly. Parallel assessment of both AM and item memory, as well as related memory phenomena (e.g., proactive/retractive interference), would provide a more comprehensive picture. This is important since previous research with patients exhibiting hippocampal lesions detected the presence of either an isolated deficit in AM task performance [28] or impairment in both, AM and item memory [29].

Here, in the randomized sham-controlled study we aimed to investigate the cumulative effects of anodal stimulation over PPC on AM performance in the context of other memory functions. Healthy young adults were allocated to either anodal or sham groups and both groups received stimulation over three consecutive days. We compared the performance of the anodal stimulation group to that of participants receiving sham stimulation at four different timepoints: pretest, immediately after the first stimulation session, one day (posttest), and five days after the third stimulation session (follow-up). To comprehensively assess both global and function-specific modulation effects, besides AM performance, we also measured item memory (as an indicator of overall episodic memory), as well as immediate and delayed verbal memory performance, together with proactive and retroactive interference. As a control, cognitively and functionally unrelated verbal fluency task was measured.

## Methods

### Participants

**Fig 1:**
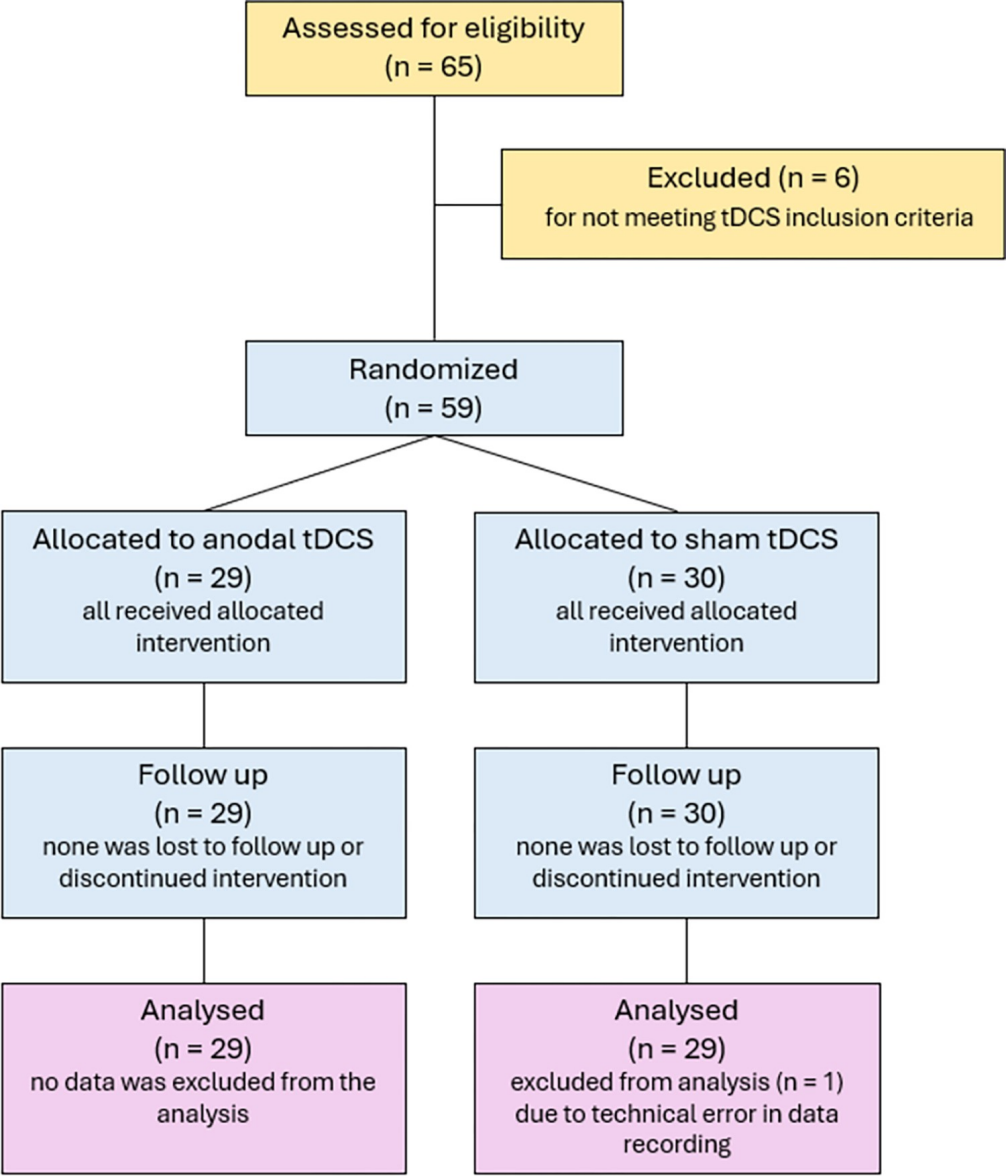
CONSORT flow diagram of study recruitment.

A total of 59 healthy young adults took part in the study (*M*_*age*_ = 21.54, *SD* = 2.5, range 18–28 years). All participants were right-handed, native speakers, with normal or corrected-to-normal vision, and fulfilled the standard tDCS inclusion criteria [30] with none reporting a history of psychiatric or neurological conditions, acute or chronic skin conditions, nor the use of psychoactive substances and medication. Based on the *a priori* power analysis in G*Power 3 [31], the minimal sample size of 58 participants (29 per group) was required to detect medium effects size (*f* = 0.42), as recommended for tDCS studies for memory enhancement based on average effect size in single-session studies [32] with the power of 0.95, at the alpha level of 0.05, and correlation between the measures of 0.50. which allows between-group comparisons at each time point independently (for power analysis across different statistical tests see OSF). The participants were included in the study on a rolling basis until the power-based target sample size was met (Fig 1). The study was approved by the Ethics Committee of the Department of Psychology, Faculty of Humanities and Social Sciences, University of Zagreb (approval number EPOP-2021-014). All participants provided written informed consent at the start of their experimental sessions.

### Measures

*Face Cued Word Recall task* (FCWR) was used to measure AM performance. Twenty face-word pairs were presented sequentially for 3000 ms each and participants were instructed to pay attention and remember as many pairs as possible. Each pair comprised a black-and-white frontal face image and a word written above it. Selected words consisted of 2 or 3 syllables and had high frequency and high imaginability (e.g., lopta /*ball*/, jagoda /*strawberry*/, etc.). Following a learning block, participants advanced to the recall block where they were shown a mix of faces from the learning block (targets) along with 30 new faces (distractors) in a pre-randomized order. Participants had to recognize whether they had seen a face before and, if so, recall the word it was presented with. Participants received no feedback regarding their performance. The task involved four blocks: practice learning block, practice recall block, test learning block, and test recall block. The purpose of such a procedure was to make sure participants were familiar with the task, had multiple chances to learn the associations (as practice and test learning blocks were identical), and had experienced both passive and active learning to enhance memory encoding and transfer into long-term memory storage.

Only performance on the test recall block was used in statistical analyses and two test scores were calculated: 1) recognition score calculated as the percentage of correctly identified faces, and 2) cued recall calculated as the percentage of correctly recalled words associated with faces. Four parallel forms of the FCWR were used across the sessions in a counterbalanced order.

*Auditory Verbal Learning Test* (RAVLT) is a word learning test, encompassing five presentations of a 25-word list (Trials A1 through A5), each followed by an attempted recall. This is followed by a second 25-word list (List B; interference list recall), after which participants were asked to again recall List A (post-interference recall trial, or Trial A6). Finally, the delayed recall of List A was tested 20 minutes later (Trial A7). Four scores were calculated: total score as the sum of correctly recalled words from Trial A1 through A5; proactive interference (performance on List B subtracted from Trial A1), retroactive interference (performance on Trial A6 subtracted from Trial A5) and delayed recall as the difference between performance on Trial 7 and Trial 5 (performance on Trial A5 subtracted from Trial A7). The RAVLT were administrated in a fixed order across sessions. Since it was a secondary outcome measure, and has only three standard parallel forms, it was administrated in three measurement points omitting the 1^st^ session–see Procedure for details).

*Verbal fluency task* involves a rapid generation of words beginning with a specified letter. Participants had 60 seconds to produce as many unique words as possible starting with a given letter. Participants were instructed not to say proper nouns and words with the same roots (e.g., bake and baker). The task was administrated in a fixed order across sessions. Each session consisted of a different set of three letters: Set 1: /p/-/t/-/z/, Set 2: /s/-/v/-/l/, Set 3: /k/-/b/-/g/, Set 4: /m/-/r/-/š/. A verbal fluency score was calculated as the average number of uniquely generated words across all three letters.

### tDCS protocol

The direct current was applied using a constant current generator and electrodes (5x5cm) inserted into saline-soaked sponges (DC-STIMULATOR PLUS by NeuroConn, Ilmenau, Germany). The anode was positioned over the left PPC, that is–P3 following the International 10–20 EEG system, and the return electrode was placed on the contralateral cheek. Under the anodal condition, a constant current of 1.5 mA was administered for 20 minutes, with a gradual fade in/out of 30 seconds. In the sham condition, the same procedure was followed, except that the current was applied only for 60 seconds at the beginning and end of the treatment (gradual fade in/out). The same stimulation protocol was successfully used for AM modulation in previous studies [25, 26].

### Procedure

**Fig 2:**
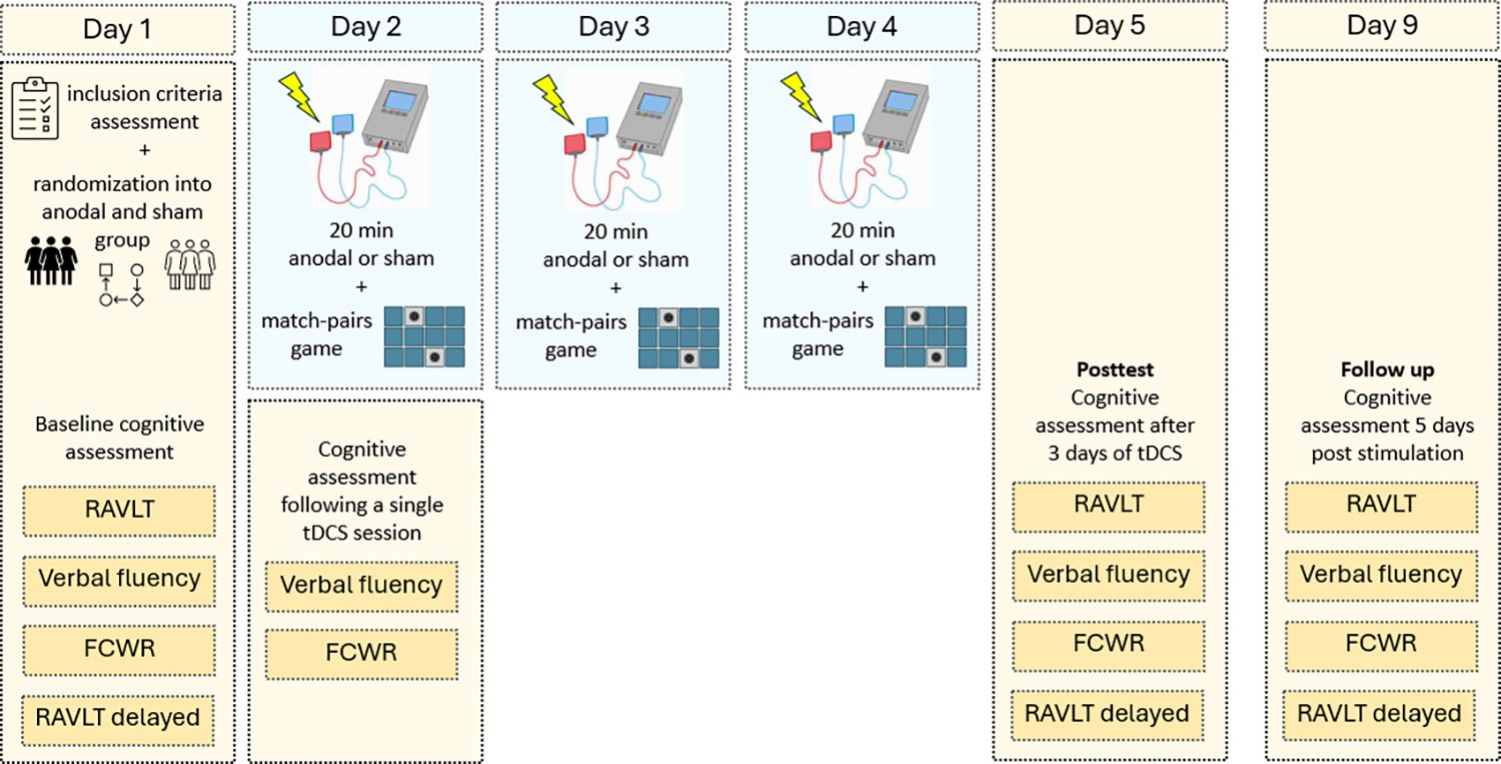
Schematic presentation of study design. Treatment participants received anodal (*n* = 29) and control participants (*n* = 29) sham stimulation over the three sessions. Measurements were taken on Day 1 (pretest), Day 2 (1^st^ session), Day 5 (posttest), and Day 9 (follow-up). Legend: RAVLT—Auditory Verbal Learning Test; FCWR—Face Cued Word Recall task.

The study design is summarized in Fig 2. On Day 1, participants underwent an initial screening to evaluate their eligibility for inclusion in the study. Those who met the inclusion criteria were randomized into a treatment group (*n* = 30) or a control (*n* = 29) group and completed Baseline tests with the tasks in the following order: RAVLT, FCWR, verbal fluency, and RAVLT (delayed recall). The order of the tasks was identical for all participants in both active and sham groups, and this particular order was chosen to ensure that the delayed recall component of the RAVLT (Trial 7) occurs at least 20 minutes after its initial RAVLT part (Trials 1–6).

Over the subsequent three sessions, participants received either anodal or sham stimulation for 20 minutes while engaging in a computerized matching-pairs game. Participants were not informed about the type of stimulation they received. Self-reports on the adverse effects of the stimulation, covering 14 different symptoms (S1 File), were collected before and immediately after each tDCS session. Each symptom was rated on a 10-point scale, and the reported intensity was interpreted as follows: 0 –no symptom, 1–2 negligible, 3–4 mild, > 4 noticeable. Following the first tDCS session (Day 2), participants completed the verbal fluency task and FCWR to assess the effects of a single stimulation session. Testing sessions were repeated on Day 5 (posttest) and Day 9 (follow-up). Parallel forms of cognitive tasks were employed across sessions to minimize practice effects, i.e. four versions of FCWR, three standard forms of RAVLT, and four fixed sets of letters for verbal fluency tasks.

### Data analysis

The statistical analysis was performed in JASP v0.19.1. The full dataset with calculated scores and the analysis outputs are available at OSF (https://osf.io/5t9rn/?view_only=13e00d69b006406aa6d3a80303ac7985).

To examine the effects of anodal stimulation on task performance mixed design analyses of variance (ANOVAs) were performed. For FCWR and verbal fluency task 2x4 ANOVAs were performed with stimulation condition (anodal / sham) as between-subject factor group and measurement timepoint (pretest / Session 1 / posttest / follow-up) as within-subject factor time. For RAVLT 2x3 ANOVAs were performed assessing the effects across three measurement points (pretest, posttest, and follow-up). We used the partial *η*_*p*_^*2*^, as a measure of effect size. To further compare data between the groups at each timepoint and reduce between-subject variability in baseline performance, we assessed group differences (independent samples t-test) in baseline-corrected performance measures, obtained by subtracting scores at Session 1, posttest, and follow-up each, from the score at the pretest, with the Cohen’s *d* as an effect size measure. All frequentist statistical tests were accompanied by the Bayes factors (BF_10_) to evaluate the level of evidence towards H1/H0 were used for these comparisons. The Lee and Wagenmakers’ classification scheme for interpreting BF10, as adopted in JASP, was used, i.e. values higher than 1 and below 3 indicate "anecdotal" evidence towards H1; values between 3 and 10 suggest "moderate" evidence; values from 10 to 30 signify "strong" evidence for H1. Conversely, BF10 lower than 1 shows that the evidence supports H0. Values lower than 1 and greater than 0.33 denote "anecdotal" support for H0; BF10 between 0.33 and 0.1 indicates "moderate" evidence; a range from 0.1 to 0.033 reflects "strong" evidence; in favor of the H0. In addition to BF10, for repeated measures ANOVA the Bayes factor for inclusion (BF_incl_), which quantifies how much more likely the data are under models that include the effect of interest compared to models that do not, was reported serving as overall evidence for the effect [33]. Post-hoc comparisons were carried out using the Bayesian t-test with corrected posterior odds. Finally, independent samples t-tests with Bonferroni correction for multiple comparisons were used to assess differences in baseline performance and unpleasantness of stimulation between anodal and sham conditions.

## Results

Across all 177 sessions, the stimulation was well tolerated, and no serious adverse effects were recorded. No differences between active tDCS and sham group were observed in any of the reported sensations (all *ps* > .11), except for the prickling/tingling sensation under the electrodes (Sham: *M* = 1.60, *SD* = 0.65, anodal tDCS: *M* = 2.27, *SD* = 1.22; *t*_*(56)*_ = 2.63, *p* = .011, *d =* 0.278). However, the average ratings were very low for both groups, with only three participants in the anodal tDCS group rating the burning sensation with a score of 4 or higher, and only one participant rated the itching sensation above 4. None of the participants experienced sensations that were intense, concerning, or significant enough to consider quitting the experiment.

**Fig 3:**
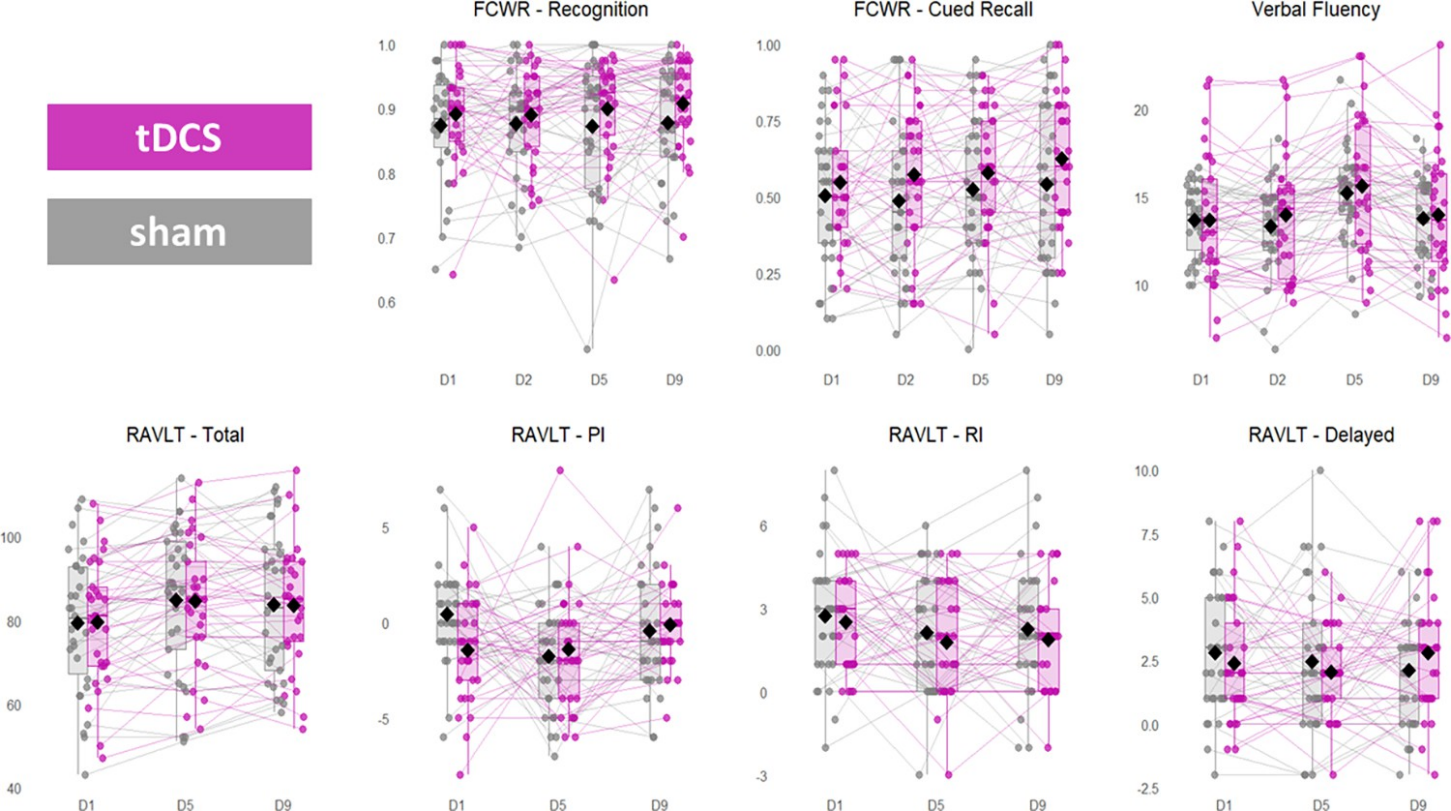
Performance across outcome measures in anodal tDCS (magenta) and sham group (gray) at different time points. Outcomes are presented on separate plots, with scores on y-axes, and timepoints on x-axes: D1 –pretest, D2 –following 1st tDCS session, D5 –posttest, D9 –follow-up. FCWR—Face Cued Word Recall task; RAVLT—Auditory Verbal Learning Test; PI–proactive interference; RI–retroactive reference. Dots represent individual data points (i.e., individual participants’ scores at each time point), diamonds indicate the mean for each group at each time point, and non-parametric indicators: interquartile range Q1-Q3 (box), median (line), range (whisker).

Descriptive statistics across all outcome measures for both anodal and sham groups are presented in Table 1. At the baseline (pretest), a significant difference between groups was observed only for the RAVLT proactive interference score (*t* (56) = 2.58, *p* = .012, *d = 0*.28). However, the difference was not statistically significant after the Bonferroni correction for multiple comparisons. All other measures were equal across groups at baseline (paired t-test, all *p* > .30). At the baseline as well as at all other timepoints, a significant variability, i.e. individual differences in performance, was observed in both groups across all measures (Fig 3).

**Table 1:** Descriptive statistics across groups and time points.

	Pretest		1^st^ session		Posttest		Follow-up	
	Sham	Anodal	Sham	Anodal	Sham	Anodal	Sham	Anodal
FCWR recognition (proportion)	.87 (.09)	.89 (.08)	.88 (.09)	.89 (.07)	.87 (.11)	.90 (.08)	.88 (.09)	.91 (.07)
FCWR recall (proportion)	.50 (.23)	.55 (.22)	.49 (.26)	.57 (.24)	.53 (.24)	.58 (.22)	.54 (.28)	.63 (.22)
RAVLT total	78.5 (16.7)	79.5 (15.3)	-	-	84.9 (17.7)	84.6 (15.0)	83.8 (17.7)	83.6 (15.0)
RAVLT proactive	0.4 (2.6)	-1.5 (2.9)	-	-	-1.8 (2.8)	-1.4 (3.2)	-0.5 (3.3)	-0.1 (2.2)
RAVLT retroactive	2.7 (2.3)	2.5 (1.7)	-	-	2.2 (2.2)	1.8 (2.0)	2.3 (2.4)	1.9 (1.8)
RAVLT delayed	2.7 (2.5)	2.4 (2.4)	-	-	2.5 (3.0)	2.0 (1.8)	2.1 (2.3)	2.8 (2.6)
Verbal fluency	13.6 (2.0)	13.7 (3.7)	13.3 (2.6)	14.0 (3.6)	15.2 (2.7)	15.6 (3.9)	13.8 (2.5)	14.0 (3.9)

Note: Means and standard deviations are presented in cells–M(SD); RAVLT—Auditory Verbal Learning Test; FCWR—Face Cued Word Recall task. Empty cells reflect no RAVLT assessment following the 1^st^ session.

### FCWR task

Separate 2x4 ANOVAs were conducted for recognition and cued recall scores from the FCWR task. None of the analyses revealed significant main effects of the time or group, nor any interaction effects (Table 2). This was supported by Bayesian repeated measures ANOVA. Namely, for the recognition score, we found strong evidence to exclude the model with time (BF_incl_ = 0.02), anecdotal evidence to exclude the model with group (BF_incl_ = 0.39), and very strong evidence to exclude their interaction (BF_incl_ < 0.01). Similarly, for cued recall score, strong evidence to exclude the model with time (BF_incl_ = 0.19), anecdotal evidence to exclude group effect (BF_incl_ = 0.44), and very strong evidence to exclude their interaction (BF_incl_ = 0.02) was found.

**Table 2:** The results of ANOVA (main effect of group and time, and group x time interaction), across all outcome measures.

			*F*	*df*	*p*	*η*_*p*_^*2*^
FCWR recognition^a^	Between subjects	Group (G)	1.74	1/56	.192	.030
	Within subjects	Time point (T)	0.39	2.7/149.1	.739	.007
		G x T	0.20	2.7/149.1	.877	.004
FCWR cued recall	Between subjects	Group (G)	1.76	1/56	.190	.030
	Within subjects	Time point (T)	2.05	3/168	.109	.035
		G x T	0.22	3/168	.880	.004
RAVLT total	Between subjects	Group (G)	<0.01	1/56	.970	< .001
	Within subjects	Time point (T)	9.00	2/112	< .001	.138
		G x T	0.13	2/112	.877	.002
RAVLT proactive	Between subjects	Group (G)	0.89	1/56	.349	.016
	Within subjects	Time point (T)	3.36	2/112	.038	.057
		G x T	2.80	2/112	.065	.048
RAVLT retroactive	Between subjects	Group (G)	0.61	1/56	.438	.011
	Within subjects	Time point (T)	1.74	2/112	.180	.030
		G x T	0.07	2/112	.934	.001
RAVLT delayed	Between subjects	Group (G)	<0.01	1/56	.961	< .001
	Within subjects	Time point (T)	0.34	2/112	.715	.006
		G x T	1.29	2/112	.280	.022
Verbal fluency	Between subjects	Group (G)	0.18	1/56	.671	.003
	Within subjects	Time point (T)	16.58	3/168	< .001	.228
		G x T	0.39	3/168	.758	.007

Note.

^a^ Greenhouse-Geiser sphericity correction

Additionally, independent samples t-tests, accompanied by Bayes factors (BF_10_), were conducted on baseline-corrected performance scores. These tests revealed no significant differences between groups, providing moderate evidence for the null hypothesis after Session 1 (*t*_(56)_ = 0.33, *p* = .743, *d* = .087, BF_10_ = 0.279), at posttest (*t*_(56)_ = -0.28, *p* = .777, *d* = -.075, BF_10_ = 0.275), and at follow-up (*t*_(56)_ = -0.34, *p* = .737, *d* = -.089, BF_10_ = 0.279) for recognition performance. A similar pattern of results was observed for AM cued recall performance after Session 1 (*t*_(56)_ = -0.62, *p* = .537, *d* = -.163, BF_10_ = 0.313), at posttest (*t*_(56)_ = < -0.01, *p* = 1.000, *d* < .001, BF_10_ = 0.266; and the follow-up too (*t*_(56)_ = -0.53, *p* = .602, *d* = -.138, BF_10_ = 0.299).

### RAVLT

Total, delayed, and post-interference recall scores from the RAVLT were analyzed using separate 2x3 ANOVAs. For the RAVLT total score and proactive interference, a significant main effect of time was found (see Table 2). This was corroborated by Bayesian approach, showing strong support for the model that included only the main effect of time (BF_10_ = 108.4) in predicting the RAVLT total score with very strong evidence for this effect (BF_incl_ = 76.38). More specifically, participants in both groups achieved higher total scores at the posttest (*t*_(57)_ = - 4.07, *p* < .001, BF_10,_ = 168.12) and follow-up (*t*_(57)_ = -3.04, *p* = .003, BF_10_ = 9.24) compared to the pretest. In contrast, models incorporating group (BF_incl_ = 0.33) and interaction effects (BF_incl_ = 0.12) received strong support to be rejected. For proactive interference, only anecdotal evidence for a model including time (BF_10_ = 1.48, BF_incl_ = 1.26) was found, with proactive interference being higher at posttest compared to follow-up (*t*_(56)_ = 2.55, *p* = .041, BF_10_ = 2.88). However, no differences between the anodal and sham groups were observed, nor were there any significant group x time interactions. Bayesian approach also showed strong evidence to exclude other models (all BF_incl_ < 0.31). For retroactive interference and delayed recall score no significant effects were found.

A comparison of baseline-corrected performance scores on the RAVLT revealed no difference between the anodal and sham group performances. Both posttest and follow-up baseline-corrected RAVLT total scores showed no significant effect, supported by moderate Bayesian evidence (Posttest: *t*_(56)_ = 0.48, *p* = .636, *d =* .125, BF_10_ = 0.293; Follow-up *t*_(56)_ = 0.39, *p* = .696, *d* = .103, BF_10_ = 0.284). Similarly, neither baseline-corrected scores for retroactive interference (Posttest: *t*_(56)_ = 0.29, *p* = .773, *d* = .076, BF_10_ = 0.276; Follow-up: *t*_(56)_ = 0.33, *p* = .745, *d* = .086, BF_10_ = 0.278) nor delayed recall (Posttest: *t*_(56)_ = 0.1, *p* = .923, *d* = .025, BF_10_ = 0.267; Follow-up: *t*_(56)_ = -1.23, *p* = .225, *d* = -.322 BF_10_ = 0.498) showed significant differences, with moderate to anecdotal evidence supporting the null hypothesis. Baseline-corrected proactive interference score showed a marginal difference between groups at posttest with smaller interference in the active tDCS group (*t*_(56)_ = -1.907, *p* = .062, *d* = -.501, BF_10_ = 1.192). At the follow-up, anecdotal evidence for baseline-corrected proactive interference decrease in anodal group was found (*t*_(56)_ = -2.122, *p* = .038, *d* = -.557, BF_10_ = 1.690).

### Verbal fluency task

For the verbal fluency task, the main effect of group and interaction were not statistically significant, but the main effect of time was found (Table 2). We found very strong evidence supporting the model that included the effect of time (BF_10_ = 7.6 x 10^6^, BF_incl_ = 5.2 x 10^6^). Specifically, participants in both the anodal and sham groups produced more words at the posttest compared to other measurement points (all *p* < .001; all BF_10,_ > 8378). Models with group (BF_incl_ = 0.294) and interaction effect (BF_incl_ = 0.089) received strong support to be excluded (see at OSF link for all statistics). No differences between groups were additionally found when baseline-corrected results were analyzed. All three comparisons had *p* > .268 and BF_10_ ranged from 0.272 to 0.449, providing moderate to anecdotal support for the null hypothesis.

## Discussion

In this randomized controlled study, we sought to examine the cumulative effects of anodal tDCS over the parietal cortex on AM performance within the context of other memory functions. Building on promising findings from a series of previous single-session studies [25, 26], we expected that participants receiving anodal tDCS would demonstrate enhanced AM performance, both immediately after the first stimulation session, as well as following the three consecutive days of tDCS, with sustained improvements evident during a follow-up assessment after five days.

Notably, several prior studies have explored the use of tDCS to modulate AM [36–41], but only one study assessed the effects of multiple stimulation sessions [42]. In the later study, Meinzer and colleagues, applied tDCS over the left PFC for five consecutive days while participants learned new vocabulary in an AM paradigm (object-nonwords), and showed a steeper learning curve in real tDCS group compared to the sham in a cued recall, but no effects on recognition. Our research introduced a similar multisession approach but targeted the parietal cortex, filling a gap in literature as previous studies with posterior montages used only single stimulation sessions. This approach holds particular relevance for potential clinical applications, where repeated administration of such interventions is foreseeable. The design of this study followed previous research indicating that anodal tDCS applied above the PPC could modulate AM performance [25–27]. Consistent with these earlier studies, we recruited healthy young participants, used the same cortical target and electrode montage, and applied a similar stimulation protocol. However, unlike the single-session designs of prior research, we administered stimulation over three consecutive days.

Contrary to our expectations, anodal stimulation failed to yield significant improvements in participants’ performance on AM measures when compared to those who received sham stimulation. The absence of effects was observed following one tDCS session, as well as three tDCS sessions carried out on consecutive days. Nevertheless, a minor effect was registered on the proactive interference score, indicating a possibility for the presence of some impact on memory processes.

Despite not meeting our principal expectations, the study results provided several valuable insights that will help with further exploration of this subject. Firstly, it is essential to highlight the high tolerance and minimal adverse effects associated with the stimulation, which resulted in high compliance and no dropouts. Even though this is in accordance with the general recognition of tES as safe and well-tolerated [30, 43] it is nevertheless an important issue proving the feasibility of the multisession application of this stimulation protocol in memory modulation studies.

Next, although the study was well-powered, judging by the effect sizes reported in the previous experiments, the Bayesian inference approach did not show strong evidence for the absence of effects. On the contrary, the evidence in favor of the null hypothesis was moderate at best. This is mostly due to the high within-group variability, which could be observed at the baseline, as well as at all subsequent assessments. Such variability is inevitable as it stems from individual differences in memory abilities. However, this type of variability can obscure the potential effects of tDCS, not only for formal statistical reasons, but also because people with different levels of initial performance may respond differently to stimulation [44], while one can also expect different trajectories at different performance levels in the days following the stimulation.

Differences in study design may also account for the divergence in results between our study and previous research, and provide valuable insights into the mechanisms of parietal stimulation effects on AM. Namely, previous studies used cross-over designs which are better suited for assessing single-session tDCS effect, but also have the advantage of controlling for within-group variability. Given the already discussed high variability in the participant’s performance, the parallel group design employed in this study might have been a limiting factor for detecting tDCS effects, as recently shown by a meta-study on tDCS methodology [45]. It is also to note here that the order of the tasks was the same for all participants. The methodological choice of fixed vs counterbalanced order of tasks is important whenever multiple outcomes are assessed. The fixed order allows for all participants to be assessed under the same circumstances at each time-point. In contrast, delivering tasks in counterbalanced order allows for statistical control of the task-order, but at the expense of requiring a much larger sample. Since no effects were observed on any of the tasks, the issue of task-order has very little impact on the interpretation of findings.

To further understand the results obtained here it is important to note that this study differed from the previous single-session studies in its approach to the AM assessment with FWCR task. Previous studies administered this task only after stimulation, requiring participants to memorize just 20 face-word pairs per stimulation type (i.e., in a single study arm). In contrast, we exposed participants to new blocks of 20 face-word pairs at each time point. There is a high likelihood that in this way, with each new testing session, the load on memory progressively increased effectively dampening down the potential stimulation effect. Following on this, the absence of anodal tDCS effects after the first session, contrary to earlier findings, may be explained by this increased load. Namely, in previous PPC anodal tDCS studies, the AM task was administered only once, after the tDCS. In this study, participants were exposed to one AM task (i.e., one set of face-word pairs) at the pre-test and to another (i.e., another set of face-word pairs) after the first stimulation, thus causing two different contents to compete for the same memory processes. In addition, the duration of the effect on formed AM pairs in previous studies that used a single AM task (i.e., asked participants to memorize only a single set of face-word pairs), was found to be up to 7 days following the single-session stimulation [26]. It may be that here this effect effectively interfered with the outcomes of subsequent testing sessions, particularly on the FWCR task on posttest and follow-up where increased build-up of new information pressure could have come into effect even more.

Previous single-session studies were designed to test the effects of PPC tDCS stimulation on encoding and retrieval of a specific set of information (a specific content). They showed indeed that the content was memorized better following the stimulation. However, they could not provide an answer on whether the effect generalizes to the encoding/retrieval functions in general thus allowing for subsequent similar (but not the same) contents to be memorized better too. This is a necessary piece of knowledge before considering further development of the PPC tDCS as a treatment option for people with memory impairments. To test this extended function—enhancing potential of the stimulation—it was necessary to introduce pretest, posttest, and follow-up testings with different versions of the FWCR task. Unfortunately, the current study did not provide supportive evidence of tDCS-induced function-enhancement.

Interestingly, despite all the mentioned constraints, a weak effect of stimulation on reducing proactive interference in the RAVLT task was detected after three tDCS sessions and particularly on follow-up. This result could be interpreted as an index of improved capacity for memorizing new memory items following a multi-session PPC stimulation suggesting a potential stimulation effect on memory enhancement.

An important limitation of our study that warrants consideration is that our sample comprised healthy young adults at the peak of their cognitive performance. When designing tDCS studies, researchers often face the choice between recruiting participants from a clinical or healthy population. Previous research suggests that the effects of tDCS may be more pronounced in elderly participants and clinical populations compared to young adults [46, 47]. Despite these caveats, exploring the effects of tDCS in healthy populations, same as with other potential treatment approaches, is a necessary first step before exploration of its treatment potentials in populations with memory impairments. Besides the obvious assessment of feasibility and safety of the tested approach, studies on healthy populations usually provide useful indications on the underlying mechanisms of tDCS effects. From an ethical standpoint, it is essential to consider that vulnerable populations in need of intervention and support may spend valuable time participating in research that could be devoted to interventions with known efficacy. Furthermore, our findings revealed a potential ceiling effect in the recognition task, with participants demonstrating an average performance of approximately 90%, which may have limited the detection of further tDCS-related improvements. Additionally, recognition tasks are influenced by various other cognitive processes such as probability-based decision-making, response styles, strategies, and biases [48], making them less ideal for capturing specific tDCS effects compared to cued recall.

In conclusion, our study did not find significant improvements in AM performance following cumulative offline anodal stimulation in healthy young adults. Despite closely mirroring previously investigated electrode montage and stimulation protocol, our results did not provide support for the hypothesized enhancements. While the study aimed to capture the time course of tDCS effects on AM function, multiple measurements may have interfered with the outcomes, obscuring the stimulation effects. The detection of the stimulation effects on AM in this study was already precious since participants were young well-functioning people with probably little space for further improvements, while the measured variable displayed large within-group variability. Nevertheless, and despite the essentially null results, this study provides some important insights relevant to designing studies aimed at the improvement of AM (and episodic memory functions in general). The most important of these are (a) to be mindful about the potential content interference when employing assessment at multiple timepoints or via different memory tests, (b) to have more stimulation sessions to allow neuromodulation effects to take place, and (c) to include participants with a certain level of memory difficulties to allow for improvement to be detected. In addition, personalization of the stimulation location based on neuroimaging and field modeling as well as the use of more precise and localized stimulation techniques, such as High-Density tDCS, are approaches to be explored. Future studies should address these considerations and continue exploring the effects of tDCS on AM modulation.

## Supporting information

S1 File Descriptive statistics of self-reports on the adverse effects of stimulation (average across 3 stimulation sessions, and standard deviations). (DOCX)

## References

[1] Bjekić J, Manojlović M, Filipović SR. Transcranial Electrical Stimulation for Associative Memory Enhancement: State-of-the-Art from Basic to Clinical Research. Life. 2023;13: 1125. doi: 10.3390/life13051125 37240770 PMC10223551

[2] Dahan L, Rampon C, Florian C. Age-related memory decline, dysfunction of the hippocampus and therapeutic opportunities. Prog Neuropsychopharmacol Biol Psychiatry. 2020;102: 109943. doi: 10.1016/j.pnpbp.2020.109943 32298784

[3] Grady C. The cognitive neuroscience of ageing. Nat Rev Neurosci. 2012;13: 491–505. doi: 10.1038/nrn3256 22714020 PMC3800175

[4] Old SR, Naveh-Benjamin M. Differential effects of age on item and associative measures of memory: A meta-analysis. Psychol Aging. 2008;23: 104–118. doi: 10.1037/0882-7974.23.1.104 18361660

[5] Naveh-Benjamin M, Mayr U. Age-related differences in associative memory: Empirical evidence and theoretical perspectives. Psychol Aging. 2018;33: 1–6. doi: 10.1037/pag0000235 29494173

[6] Naveh‐Benjamin M. Associative Deficit Hypothesis. 1st ed. In: Whitbourne SK, editor. The Encyclopedia of Adulthood and Aging. 1st ed. Wiley; 2015. pp. 1–5. doi: 10.1002/9781118521373.wbeaa287

[7] Cummings JL, Tong G, Ballard C. Treatment Combinations for Alzheimer’s Disease: Current and Future Pharmacotherapy Options. J Alzheimers Dis. 2019;67: 779–794. doi: 10.3233/JAD-180766 30689575 PMC6398562

[8] O’Brien JT, Holmes C, Jones M, Jones R, Livingston G, McKeith I, et al. Clinical practice with anti-dementia drugs: A revised (third) consensus statement from the British Association for Psychopharmacology. J Psychopharmacol (Oxf). 2017;31: 147–168. doi: 10.1177/0269881116680924 28103749

[9] Martínez-López S, Tabone M, Clemente-Velasco S, González-Soltero MDR, Bailén M, De Lucas B, et al. A systematic review of lifestyle-based interventions for managing Alzheimer’s disease: Insights from randomized controlled trials. J Alzheimer’s Dis. 2024; 13872877241292829. doi: 10.1177/13872877241292829 39584279

[10] Bikson M, Grossman P, Thomas C, Zannou AL, Jiang J, Adnan T, et al. Safety of Transcranial Direct Current Stimulation: Evidence Based Update 2016. Brain Stimulat. 2016;9: 641–661. doi: 10.1016/j.brs.2016.06.004 27372845 PMC5007190

[11] Elder GJ, Taylor J-P. Transcranial magnetic stimulation and transcranial direct current stimulation: treatments for cognitive and neuropsychiatric symptoms in the neurodegenerative dementias? Alzheimers Res Ther. 2014;6: 74. doi: 10.1186/s13195-014-0074-1 25478032 PMC4255638

[12] Nitsche MA, Paulus W. Excitability changes induced in the human motor cortex by weak transcranial direct current stimulation. J Physiol. 2000;527: 633–639. doi: 10.1111/j.1469-7793.2000.t01-1-00633.x 10990547 PMC2270099

[13] Antonenko D, Schubert F, Bohm F, Ittermann B, Aydin S, Hayek D, et al. tDCS-Induced Modulation of GABA Levels and Resting-State Functional Connectivity in Older Adults. J Neurosci. 2017;37: 4065–4073. doi: 10.1523/JNEUROSCI.0079-17.2017 28314813 PMC6596583

[14] Frase L, Mertens L, Krahl A, Bhatia K, Feige B, Heinrich SP, et al. Transcranial direct current stimulation induces long-term potentiation-like plasticity in the human visual cortex. Transl Psychiatry. 2021;11: 17. doi: 10.1038/s41398-020-01134-4 33414402 PMC7791098

[15] Eichenbaum H, Yonelinas AP, Ranganath C. The Medial Temporal Lobe and Recognition Memory. Annu Rev Neurosci. 2007;30: 123–152. doi: 10.1146/annurev.neuro.30.051606.094328 17417939 PMC2064941

[16] Kim K, Ekstrom AD, Tandon N. A network approach for modulating memory processes via direct and indirect brain stimulation: Toward a causal approach for the neural basis of memory. Neurobiol Learn Mem. 2016;134: 162–177. doi: 10.1016/j.nlm.2016.04.001 27066987

[17] Achim AM, Lepage M. Neural Correlates of Memory for Items and for Associations: An Event-related Functional Magnetic Resonance Imaging Study. J Cogn Neurosci. 2005;17: 652–667. doi: 10.1162/0898929053467578 15829085

[18] Mayes A, Montaldi D, Migo E. Associative memory and the medial temporal lobes. Trends Cogn Sci. 2007;11: 126–135. doi: 10.1016/j.tics.2006.12.003 17270487

[19] Wagner AD, Shannon BJ, Kahn I, Buckner RL. Parietal lobe contributions to episodic memory retrieval. Trends Cogn Sci. 2005;9: 445–453. doi: 10.1016/j.tics.2005.07.001 16054861

[20] Hermiller MS, VanHaerents S, Raij T, Voss JL. Frequency‐specific noninvasive modulation of memory retrieval and its relationship with hippocampal network connectivity. Hippocampus. 2019;29: 595–609. doi: 10.1002/hipo.23054 30447076 PMC6525080

[21] Nilakantan AS, Bridge DJ, Gagnon EP, VanHaerents SA, Voss JL. Stimulation of the Posterior Cortical-Hippocampal Network Enhances Precision of Memory Recollection. Curr Biol. 2017;27: 465–470. doi: 10.1016/j.cub.2016.12.042 28111154 PMC5302852

[22] Tambini A, Nee DE, D’Esposito M. Hippocampal-targeted Theta-burst Stimulation Enhances Associative Memory Formation. J Cogn Neurosci. 2018;30: 1452–1472. doi: 10.1162/jocn_a_01300 29916791 PMC7467684

[23] Wang JX, Rogers LM, Gross EZ, Ryals AJ, Dokucu ME, Brandstatt KL, et al. Targeted enhancement of cortical-hippocampal brain networks and associative memory. Science. 2014;345: 1054–1057. doi: 10.1126/science.1252900 25170153 PMC4307924

[24] Friedman NP, Robbins TW. The role of prefrontal cortex in cognitive control and executive function. Neuropsychopharmacology. 2022;47: 72–89. doi: 10.1038/s41386-021-01132-0 34408280 PMC8617292

[25] Bjekić J, Čolić MV, Živanović M, Milanović SD, Filipović SR. Transcranial direct current stimulation (tDCS) over parietal cortex improves associative memory. Neurobiol Learn Mem. 2019;157: 114–120. doi: 10.1016/j.nlm.2018.12.007 30553021

[26] Bjekić J, Vulić K, Živanović M, Vujičić J, Ljubisavljević M, Filipović SR. The immediate and delayed effects of single tDCS session over posterior parietal cortex on face-word associative memory. Behav Brain Res. 2019;366: 88–95. doi: 10.1016/j.bbr.2019.03.023 30880221

[27] Vulić K, Bjekić J, Paunović D, Jovanović M, Milanović S, Filipović SR. Theta-modulated oscillatory transcranial direct current stimulation over posterior parietal cortex improves associative memory. Sci Rep. 2021;11: 3013. doi: 10.1038/s41598-021-82577-7 33542344 PMC7862221

[28] Holdstock JS, Mayes AR, Gong QY, Roberts N, Kapur N. Item recognition is less impaired than recall and associative recognition in a patient with selective hippocampal damage. Hippocampus. 2005;15: 203–215. doi: 10.1002/hipo.20046 15390152

[29] Wixted JT, Squire LR. Recall and recognition are equally impaired in patients with selective hippocampal damage. Cogn Affect Behav Neurosci. 2004;4: 58–66. doi: 10.3758/cabn.4.1.58 15259889

[30] Antal A, Alekseichuk I, Bikson M, Brockmöller J, Brunoni AR, Chen R, et al. Low intensity transcranial electric stimulation: Safety, ethical, legal regulatory and application guidelines. Clin Neurophysiol. 2017;128: 1774–1809. doi: 10.1016/j.clinph.2017.06.001 28709880 PMC5985830

[31] Faul F, Erdfelder E, Lang A-G, Buchner A. G*Power 3: A flexible statistical power analysis program for the social, behavioral, and biomedical sciences. Behav Res Methods. 2007;39: 175–191. doi: 10.3758/bf03193146 17695343

[32] Bjekić J, Živanović M, Filipović SR. Transcranial Direct Current Stimulation (tDCS) for Memory Enhancement. J Vis Exp. 2021; 62681. doi: 10.3791/62681 34605816

[33] Hinne M, Gronau QF, Van Den Bergh D, Wagenmakers E-J. A Conceptual Introduction to Bayesian Model Averaging. Adv Methods Pract Psychol Sci. 2020;3: 200–215. doi: 10.1177/2515245919898657

[34] JASP Team. JASP. 2024. Available: https://jasp-stats.org

[35] Lee MD, Wagenmakers E-J. Bayesian Cognitive Modeling: A Practical Course. 1st ed. Cambridge University Press; 2014. doi: 10.1017/CBO9781139087759

[36] Brunyé TT, Smith AM, Horner CB, Thomas AK. Verbal long-term memory is enhanced by retrieval practice but impaired by prefrontal direct current stimulation. Brain Cogn. 2018;128: 80–88. doi: 10.1016/j.bandc.2018.09.008 30414699

[37] Gaynor AM, Chua EF. tDCS over the prefrontal cortex alters objective but not subjective encoding. Cogn Neurosci. 2017;8: 156–161. doi: 10.1080/17588928.2016.1213713 27417530 PMC5505562

[38] Huang Y, Mohan A, McLeod SL, Luckey AM, Hart J, Vanneste S. Polarity-specific high-definition transcranial direct current stimulation of the anterior and posterior default mode network improves remote memory retrieval. Brain Stimulat. 2021;14: 1005–1014. doi: 10.1016/j.brs.2021.06.007 34182233

[39] Leshikar ED, Leach RC, McCurdy MP, Trumbo MC, Sklenar AM, Frankenstein AN, et al. Transcranial direct current stimulation of dorsolateral prefrontal cortex during encoding improves recall but not recognition memory. Neuropsychologia. 2017;106: 390–397. doi: 10.1016/j.neuropsychologia.2017.10.022 29056368

[40] Marián M, Szőllősi Á, Racsmány M. Anodal transcranial direct current stimulation of the right dorsolateral prefrontal cortex impairs long-term retention of reencountered memories. Cortex. 2018;108: 80–91. doi: 10.1016/j.cortex.2018.07.012 30142573

[41] Matzen LE, Trumbo MC, Leach RC, Leshikar ED. Effects of non-invasive brain stimulation on associative memory. Brain Res. 2015;1624: 286–296. doi: 10.1016/j.brainres.2015.07.036 26236022

[42] Meinzer M, Jähnigen S, Copland DA, Darkow R, Grittner U, Avirame K, et al. Transcranial direct current stimulation over multiple days improves learning and maintenance of a novel vocabulary. Cortex. 2014;50: 137–147. doi: 10.1016/j.cortex.2013.07.013 23988131

[43] Bjekić J, Živanović M, Stanković M, Paunović D, Konstantinović U, Filipović SR. The subjective experience of transcranial electrical stimulation: a within-subject comparison of tolerability and side effects between tDCS, tACS, and otDCS. Front Hum Neurosci. 2024;18: 1468538. doi: 10.3389/fnhum.2024.1468538 39507062 PMC11537871

[44] Wu D, Zhang P, Liu N, Sun K, Xiao W. Effects of High-Definition Transcranial Direct Current Stimulation Over the Left Fusiform Face Area on Face View Discrimination Depend on the Individual Baseline Performance. Front Neurosci. 2021;15: 704880. doi: 10.3389/fnins.2021.704880 34867146 PMC8639859

[45] Santander T, Leslie S, Li LJ, Skinner HE, Simonson JM, Sweeney P, et al. Towards optimized methodological parameters for maximizing the behavioral effects of transcranial direct current stimulation. Front Hum Neurosci. 2024;18: 1305446. doi: 10.3389/fnhum.2024.1305446 39015825 PMC11250584

[46] Brunoni AR, Vanderhasselt M-A. Working memory improvement with non-invasive brain stimulation of the dorsolateral prefrontal cortex: A systematic review and meta-analysis. Brain Cogn. 2014;86: 1–9. doi: 10.1016/j.bandc.2014.01.008 24514153

[47] Cespón J, Rodella C, Rossini PM, Miniussi C, Pellicciari MC. Anodal Transcranial Direct Current Stimulation Promotes Frontal Compensatory Mechanisms in Healthy Elderly Subjects. Front Aging Neurosci. 2017;9: 420. doi: 10.3389/fnagi.2017.00420 29326582 PMC5741680

[48] Osth AF, Jansson A, Dennis S, Heathcote A. Modeling the dynamics of recognition memory testing with an integrated model of retrieval and decision making. Cognit Psychol. 2018;104: 106–142. doi: 10.1016/j.cogpsych.2018.04.002 29778777

