# Supplementary material for: Multi-session tDCS over the posterior parietal cortex and associative memory
Source: PLoS One. 2025 Jan 30;20(1):e0318593. doi: 10.1371/journal.pone.0318593 (PMC11781686; doi:10.1371/journal.pone.0318593)
Supplement: S1 File — (DOCX) [file pone.0318593.s001.docx]

**Supplementary material A. Descriptive statistics of self-reports on the adverse effects of stimulation (average across 3 stimulation sessions, and standard deviations).**

|  | Before stimulation | | After stimulation | |
| --- | --- | --- | --- | --- |
|  | Sham | Anodal | Sham | Anodal |
| Headache | 1.6 (0.8) | 1.4 (0.7) | 1.6 (0.8) | 1.5 (0.9) |
| Neck Pain | 1.4 (0.7) | 1.3 (0.6) | 1.5 (0.9) | 1.3 (0.6) |
| Back Pain | 1.5 (1) | 1.5 (1.1) | 1.5 (1.1) | 1.5 (1.1) |
| Blurry Vision | 1.1 (0.4) | 1.1 (0.2) | 1.1 (0.4) | 1.1 (0.2) |
| Skin Irritation | 1.2 (0.5) | 1.2 (0.8) | 1.5 (0.8) | 1.9 (1.3) |
| Prickling/Tingling Sensation | 1.0 (0) | 1.1 (0.4) | 1.6 (0.7) | 2.3 (1.2) |
| Itching | 1.2 (0.4) | 1.1 (0.3) | 1.6 (0.7) | 2.0 (1.2) |
| Increased Heart Rate | 1.3 (0.6) | 1.5 (0.9) | 1.1 (0.4) | 1.2 (0.5) |
| Burning Sensation | 1.0 (0.1) | 1.1 (0.2) | 1.3 (0.5) | 1.4 (0.7) |
| Dizziness | 1.1 (0.3) | 1.2 (0.6) | 1.1 (0.3) | 1.0 (0.1) |
| Hot Flashes | 1.0 (0.1) | 1.1 (0.2) | 1.1 (0.2) | 1.1 (0.3) |
| Acute Mood Swings | 1.1 (0.2) | 1.1 (0.6) | 1.1 (0.2) | 1.1 (0.3) |
| Tiredness | 2.7 (1.4) | 2.6 (1.5) | 2.8 (1.7) | 2.6 (1.4) |
| Anxiety | 1.4 (0.6) | 1.7 (1) | 1.3 (0.5) | 1.5 (0.6) |
